# Clinical Outcome, Socioeconomic Status and Psychological Constrains of Patients Undergoing Preimplantation Genetic Testing (PGT) in Northern Greece

**DOI:** 10.3390/medicina58101493

**Published:** 2022-10-20

**Authors:** Antonios Patrikiou, Alexandra Papadopoulou, Christos Noutsos, Panagiotis Tzekis, Nikolaos Koios, Ioannis Kourempeles, George Anifandis, Antonia Sioga, Grigoris Grimbizis, Basil C. Tarlatzis, Katerina Chatzimeletiou

**Affiliations:** 1 Unit for Human Reproduction, 1st Department of Obstetrics & Gynaecology, Aristotle University Medical School, Papageorgiou General Hospital, 56403 Thessaloniki, Greece; 2 Biology Department, SUNY College at Old Westbury, Old Westbury, NY 11731, USA; 3 Department of Information and Electronic Engineering, International Hellenic University, Thermi, 57001 Thessaloniki, Greece; 4 University Ecclesiastical Academy of Thessaloniki, 54250 Thessaloniki, Greece; 5 Dogmatics Theology, School of Theology, Faculty of Theology, Aristotle University of Thessaloniki, 54124 Thessaloniki, Greece; 6 Department of Obstetrics and Gynecology, School of Health Sciences, Faculty of Medicine, University of Thessaly, 41200 Larisa, Greece; 7 Laboratory of Histology and Embryology, Aristotle University Medical School, 54124 Thessaloniki, Greece

**Keywords:** preimplantation genetic testing, monogenic disorders, aneuploidy, PGT-M/PGT-A, physical and psychological pain, bioethics

## Abstract

*Background and objectives*: Preimplantation genetic testing (PGT) offers patients the possibility of having a healthy baby free of chromosomal or genetic disorders. The present study focuses on the application of PGT for patients located in Northern Greece, investigating their clinical outcomes, their motives, and their overall physical and emotional experience during the treatment, in association with their socioeconomic background. *Materials and Methods*: Couples who underwent PGT for a monogenic condition (PGT-M, *n* = 19 cycles) or aneuploidy (PGT-A, *n* = 22 cycles) participated in the study. Fertilization, implantation, and pregnancy rates were recorded for all cycles. The couples were asked to fill in a questionnaire about the consultation they had received prior to treatment, their sociodemographic information, and the psychological impact PGT had on both the female and male partner. *Results*: The fertilization, implantation, and ongoing pregnancy rates for the PGT-M and PGT-A cycles were 81.3%, 70.6%, and 52.9%, and 78.2%, 64.3%, and 57.1%, respectively. Females experienced more intense physical pain than their male partners while psychological pain was encountered by both partners and occasionally in higher instances in males. No typical socioeconomic background of the patients referred for PGT in Northern Greece was noticed. *Conclusion*: PGT is an attractive alternative to prenatal diagnosis (PND), aiming to establisha healthy pregnancy by identifying and avoiding the transfer of chromosomally or genetically abnormal embryos to the uterus. Although the benefits of PGT were well-received by all patients undergoing the procedure, psychological pain was evident and especially prominent in patients with a previous affected child or no normal embryos for transfer. Holistic counseling is of utmost importance in order to make patients’ experience during their journey to have a healthy baby less emotionally demanding and help them make the right choices for the future.

## 1. Introduction

In vitro fertilization (IVF) involves the collection of eggs from the ovary, their fertilization in the laboratory, and the transfer of the developing embryos to the woman’s uterus [1]. However, despite the huge advancements in the field of assisted reproduction, the success rates of IVF remain low, mainly because a large proportion of embryos are either unable to reach the blastocyst stage and implant, or result in miscarriage. One possible cause for this is the high incidence of chromosomal abnormalities found at the early stages of preimplantation development [2,3,4,5,6,7,8,9]. Preimplantation genetic testing for aneuploidy (PGT-A) has therefore been proposed as a solution that allows the diagnosis of chromosomal abnormalities in embryos before implantation to the uterus [10,11]. PGT-A is offered to patients of advanced maternal age (AMA) ≥ 35 years, repeated implantation failure (RIF) > 3, repeated miscarriages (RM) > 2, previous pregnancy with a chromosomally abnormal fetus irrespective of maternal age, and in cases of severe male factor infertility including non-obstructive azoospermia (NOA) and oligoasthenoteratozoospermia (OAT) [9,10,11,12,13,14,15].

The method was first introduced in 1990 as a diagnostic tool to avoid the inheritance of an X-linked disease in male offspring [16]. Since then, the method has been successfully applied for the diagnosis of monogenic disorders (PGT-M), chromosomal translocations (PGT-SR),late-onset diseases includinginherited cancers, and for Human Leukocyte Antigens (HLA) matching to identify HLA-compatibleembryos that can save the life of family members (usually an affected child) through umbilical cord blood stem cell transplantation and future bone marrow transplantations [11,17,18,19].

The most widely used embryo biopsy strategies for PGT are: (a) cleavage stage biopsy on day 3 followed by aspiration of a single blastomere and fresh embryo transfer on day 5, and (b) blastocyst stage biopsy on day 5 followed by aspiration of 5–10 trophectoderm (TE) cells, immediate vitrification of the blastocyst post biopsy, and transfer after warming at a future cycle, in a synchronized well-prepared endometrium [13,14,16,20,21,22].

Although the benefits of PGT in the establishment of healthy pregnancies and the avoidance of a potential termination are well-received, patients undergoing PGT often demonstrate signs of psychological burden which may remain up to 3 years after the procedure. Uncertainty of outcome, lack of in-depth information or understanding of the provided information, physical pain, gender differences, socioeconomical background, religious beliefs, ethical or moral perceptions, unrealistic expectations, and cost are crucial factors affecting the overall psychology and decision making [23,24,25,26,27,28,29,30,31,32,33,34,35].

The aims of the present study were as follows.

1. To give an insight into the motives and clinical outcomes of couples undergoing PGT in Northern Greece.
2. To investigate the patients’ profiles with respect to their socioeconomic background, educational level, and emotional experience during the process, as assessed by questionnaires.

## 2. Methods

The study was performed between 2017 and 2020. Clinical data were collected from the IVF units Biogenesis and Fertilia by Genesis, where couples underwent preimplantation genetic testing for monogenic disorders (PGT-M, 19 cycles) and aneuploidies (PGT-A, 22 cycles). Embryo biopsies were performed according to Chatzimeletiou et al., 2021; 2005 [13,20]. Embryos were removed from culture on day 3 and placed in a drop of calcium/magnesium-free medium (Origio, Malov, Denmark) under oil (Origio, Malov, Denmark) and immobilized by suction using a holding pipette (Humagen—Origio Malov, Denmark). A hole in the zona was created with the aid of the Saturn laser (RI; Bickland Industrial Park, Falmouth, UK) or the Lycos-DTS laser (Hamilton Thorne, Beverly, MA, USA) and a blastomere was aspirated using a biopsy pipette (COOK Medical, Bloomington, IN, USA). The blastomere was placed in a PCR tube in Ca^2+^/Mg^2+^-free phosphate buffered saline (PBS) (Gibco; Grand Island, NY, USA) and analyzed for all chromosomes by array-Comparative Genome Hybridisation(CGH) (PGT-A) or for mutation analysis by PCR (PGT-M), respectively. The analytical validity of the PGT-M results was recorded on the genetic analysis report provided by the genetics lab that performed the analysis and ranged between 95–99%. It was also noted that due to the risk of allele drop out and mosaicism, follow-up of the pregnancies established by invasive prenatal testing is recommended. For the PGT-A results it was also noted that, due to the risk of mosaicism, the accuracy of results is around 90% based on the current literature and therefore follow-upby prenatal testing is recommended [4,11]. The culture of all embryos up to day 5 in Irvine(FUJIFILM Irvine Scientific- Santa Ana, CA, USA) or SAGE (Malov, Denmark) culture medium followed. One to a maximum of two embryos that were diagnosed as normal after PGT-A/M were transferred to the uterus on day 5 and any surplus normal embryos were vitrified for clinical purposes. The patients were also asked about the fate of the embryos that were diagnosed as affected, following PGT-M, or chromosomally abnormal, following PGT-A, and signed whether they consent to donate them for research or destroy them.

A questionnaire was handed out to couples undergoing preimplantation genetic testing (PGT-M, PGT-A). The questionnaire was divided into three parts and addressed issues regarding the process of PGT and patient experience (Parts A and B), as well as socioeconomic and demographic parameters (Part C). Twenty couples returned the answered questionnaires for evaluation anonymously. Data were collected and statistically analyzed with statistical significance set at 0.05 (*p* < 0.05). Linear and binary regression analysis was used to explore both the physical and emotional impact of PGT on the patients. 

### Ethical Approval

Legislation in Greece (Law no: 3305/2005, ΦΕΚ 4875/Β/29-12/17) allows PGT for the following indications: (1) PGT-A for patients with AMA, RM, RIF, and previous aneuploid pregnancy; (2) PGT-SR for structural rearrangements including reciprocal and Robertsonian translocations; (3) PGT-M for monogenic disorders and X-linked diseases; (4) hereditary cancers (families with severe history only); and (5) HLA-matching. Ethical approval by the Aristotle University Medical School was obtained for this study (No: 388/8/22.2.2017) and for PGT and research on human embryos (No: 1.31/21/11/2018).

## 3. Results

### 3.1. Clinical Data

The clinical data for PGT-M (*n* = 19 cycles) and PGT-A (*n* = 22 cycles) are shown in Table 1 and Table 2, respectively, and the couples’ indications for PGT-M and PGT-A are shown in Table 3. In the PGT-M group, 2/19 cycles (10.5%) had no normal or carrier embryos for transfer, while in the PGT-A group, 8 cycles (38.1%) had no normal/euploid embryos for transfer. The fertilization, implantation (+hCG/ET), and ongoing pregnancy/ET rates for the PGT-M cycles were 81.3% (174/214), 70.6% (12/17), and 52.9% (9/17), and for the PGT- A cycles, 78.2% (176/225), 64.3% (9/14), and 57.1% (8/14), respectively (Table 1 and Table 2). In the PGT-M group, one twin pregnancy was complicated by premature rapture of amniotic membranes and was lost, and another pregnancy with a normal fetus for cystic fibrosis was terminated after being diagnosed with Trisomy 14 during prenatal diagnosis by chorionic villus sampling. In total in the PGT-M group, 9 pregnancies went to term (8 singleton and 1 twin) resulting in 10 healthy babies born (8 males and 2 females), while in the PGT-A group, 8 pregnancies went to term (6 singleton and 2 twin) resulting in 10 healthy babies born (6 males and 4 females).

### 3.2. Questionnaire Results

**Figure 1:**
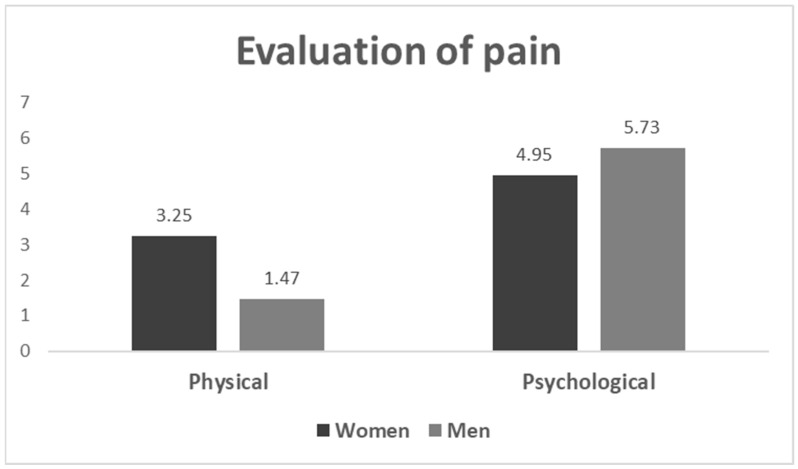
Evaluation of pain in patients undergoing PGT.

Twenty couples returned the questionnaires of which eight underwent PGT-M (12 cycles) and the remaining twelve PGT-A(15 cycles). In Part A, the couples were asked to answer questions about the purpose of their choice to try assisted reproduction services and to undergo PGT, as well as whether they had received consultation or guidance towards this decision and by whom. The majority of couples stated that their gynecologist suggested IVF with PGT (19/20 95%).Part B included questions regarding the sources of information on IVF and PGT and the majority of couples again stated that their gynecologist (private sector) gave them most information and referred them to an embryologist/geneticist to provide them with all the technical information regarding embryo biopsy and PGT(18/20, 90%). Moreover, Part B was designed to provide insight into the physical and emotional impact the entire process had on the couples, as well as whether there was a distinction in the levels of pain felt between the two sexes. The couples were asked to evaluate the stress inflicted on them during the assisted reproduction experience ona scale from zero (0) to ten (10), both for physical and psychological pain. Physical pain was described as generally low but noticeably increased in females, whereas psychological pain ranged ona higher scale and was interestingly increased in males (Figure 1). The binary logistic regression analysis indicated no significant difference in the experience of psychological pain between male and female participants (*p* = 0.056), whereas a significant difference was recorded in the experience of physical pain (*p* = 0.042) (Table 4).

The sociodemographic determinants of Part C included age, education, origin, employment, religious affiliations, and income (Table 5). More specifically, the mean age of the male participants was 40.9 years, whereas the mean age of the female ones was 38.0 years. Age as a factor generally played a major role in the couples’ decision-making about opting for preimplantation genetic testing (PGT-A). Regarding the couple’s origin, only a mere 5% of the participants were not of Greek origin. In terms of education, the majority of them had a university degree (48.6%) and a further (14.3%) had postgraduate studies with Master of Sciences or Doctor of Philosophy degrees; 34.3% had received secondary education and a minority of patients (2.9%) had received primary education only. There were variations in the employment status of both male and female participants, the majority of whom were working either in the private (48.6%) or the public sector (25.7%) and only 2.9% being unemployed. Finally, religious affiliations were of significant importance. All males and most of the females (90%) had ties to religion. Regarding couple’s annual earned income, 11.4% of the households earned less than 10,000 € per year, 11.4% earned 10,000 €–19,999 €, 34.3% earned 20,000 €–29,999 €, 28.6% earned 30,000 €–39,999 €, and 8.6% earned 40,000 €–49,999 € (Table 5).

The couples were asked if they consent to the donation of embryos identified as abnormal following PGT for research purposes, prior to their destruction. 90% of the couples (18/20) allowed the donation of their chromosomally abnormal and affected embryos for research.

## 4. Discussion

This study investigated both the clinical outcomes and impact of PGT on couplesin Northern Greece, with respect to their socioeconomic background, educational level, and emotional experience during the process, as assessed by questionnaires.The main reason for choosing PGT-M was the prevention of pregnancy termination or birth of a child that would suffer from a severe genetic disease, and in the case of HLA-matching to create a saviour sibling. For PGT-A the primary cause was the establishment of a healthy pregnancy and avoidance of miscarriage or termination of a pregnancy with a chromosomally abnormal fetus. Religion, patient’s ethical status regarding embryo creation, and patient’s experience of a previous affected/aneuploid pregnancy played a key role in the decision to undergo PGT.These results are in agreement with other studies reporting that patients with a sick child or previous experience of termination were more keen to use the technology in order to have a healthy baby [24,25,26].

Most of the PGT-M cycles performed were for beta-thalassemia (Table 3). In total, 9 PGT-M cycles were performed for b-thalassemia of which 6/9 had +vehCG/ET, but one twin pregnancy was complicated by premature rapture of amniotic membranes, leading to only five singleton term pregnancies and the birth of five boys. One patient had 2 PGT-M cycles forb-thalassemia and microdrepanocytosis, of which one had ET with no positive hCG result, and another patient underwent a successful PGT-M cycle for b-thalassemia and sickle-cell anemia, which led to the birth of a boy. B-thalassemia is unevenly distributed among different geographical regions, with high prevalence in the Mediterranean countries, particularly Greece, hence the alternative name Mediterranean anaemia [36]. Beta thalassemia has been associated with *Plasmodium falciparum*, responsible for the deadly form of malaria. Carriers of the mutated hemoglobulin are believed to show resistance to the plasmodium through cellular and/or immune-related mechanisms [37]. As a result, during past high incidences of malaria in the Mediterranean, carriers survived and prevailed in the regions, transmitting the gene through generations.

One couple in our study underwent two PGT-M cycles with HLA typing for chronic granulomatous disease (CGD).The couple had an affected boy with CGD inherited by the mother who was a carrier of mutation c.674 + 4A > T in gene CYBB in chromosome X and needed stem cell transplantation but both parents were not compatible donors. As a result, the couple decided to undergo PGT-M with HLA matching in order to find non-affected by CGD/HLA-matched embryos for transfer. Unfortunately, none of the ten biopsied embryos in this first cycle were HLAmatched with the affected child. Five were affected, three carriers, and two normal for CGD. The couple decided to vitrify the two normal and three carrier embryos and proceed to a second cycle in the hopes that HLA-matched embryos would be available this time to save the life of their son. Indeed, in the second cycle 14 embryos were biopsied and the results showed four normal HLA-matched, two carrier HLA-matched, fivecarrier not HLA-matched, and three affected not HLA-matched embryos. One normal HLA-matched embryo was transferred to the mother’s uterus leading to the establishment of a normal pregnancy and the birth of a healthy baby girl. All remaining HLA-matched normal or carrier embryos and the normal or carrier not HLA-matched embryos were vitrified. Cord blood stem cells were collected at birth and a year later cord blood stem cellstransplantation and bone marrow stem cells transplantation from the HLA-matched savior sister saved the life of her affected brother.

Embryo cryopreservation is generally widely used in the European Union (EU), with minor exceptions like in Poland, where it is still under legal debate, and Germany, where cryopreservation is legal at the 2PN stage and prohibited at the cleavage/blastocyst stage unless there is a medical emergency. In Greece, embryos can remain cryopreserved for 5 years with the possibility of extension to another 5 years during which time patients have the option to transfer them should they wish to have another child. If they do not wish to use them for their own clinical purposes, the patients may consent to donate their cryopreserved embryos to other childless couples or for research purposes or sign for destruction. Complex ethical and legal issues may also arise related to disposition of cryopreserved gametes and embryos in the event of divorce or death of one of the prospective parents. According to the legislation in Greece, both men and women can use their own cryopreserved gametes after divorce without the need for the husband or wife to sign a consent form. However, embryos can only be used after informed signed consent of both spouses. The same applies for the use after death. In the event that one does not sign, the embryos can remain cryopreserved and then either be used for clinical purposes or destroyed or donated for research depending on the decision/act of the National Committee of Assisted Reproduction [Law no 4958 ΦΕΚ21/7/2022]. Reflecting society’s difficulties in defining the moral, ethical, and legal status of human embryos, such cases can become legal battle grounds.

Of special interest is a PGT-A case of a woman with repeated miscarriages that developed Asherman Syndrome. Although chromosomally normal embryos were diagnosed after PGT-A, pregnancy could not be sustained due to the affected endometrium. Therefore, this patient chose to use a surrogate mother who successfully carried out the pregnancy and gave birth to a healthy baby boy.

Gestational surrogacy can be commercial or non-commercial (altruistic), depending on whether the surrogate mother is entitled to a financial profit from the gestation or not. Enforcement of Medically Assisted Reproduction (Law 3305/2005) in Greece stipulates altruistic surrogacy, as a part of assisted reproduction, but legislation on the matter differs among the rest of the European countries. In Finland, Norway, Sweden, Austria, France, Germany, Iceland, Bulgaria, Hungary, Lithuania, Serbia, Slovenia, Slovakia, Spain, Italy, and Switzerland, any altruistic or commercial surrogacy arrangements are prohibited (European Society of Human Reproduction and Embryology), contrary to Georgia, Ukraine, and Russia. Commercial surrogacy alone is illegal in Belgium, the Netherlands, Portugal, and the United Kingdom. Surrogacy, in both its forms, has been met with debate; commercial surrogacy raises ethical concerns since it objectifies the surrogate mother for the fulfilment of a cause that does not contribute to her own well-being [38]. Additionally, the cost of non-altruistic surrogacy is a privilege of the minority that can afford it, discriminating against aspiring parents with less financial income [39]. As far as altruistic surrogacy is concerned, it has been surrounded with controversy due to the lack of insight regarding the surrogate’s true motivation to selflessly give birth to another person’s offspring; it is believed that some women opt for surrogacy to deal with personal guilt due to terminating pregnancy in the past or placing their child up for adoption [40]. Finally, opponentsof surrogacy in all its forms advocate that both the biological and psychological bond formed during pregnancy between the surrogate and the baby eventually has to be broken, increasing the chances of depression or other emotional burdens in the postpartum period for the gestational mother [41].

The variations in the social, educational, and economical background of the couples included in the present study indicate that there is no typical profile for aspiring parents resorting to IVF and PGT; their common ground, despite religious affiliations, income-deriving hesitations, and the ever-present possibility of treatment failure, is their strong motivation for childbearing. One exemplary case is a couple that underwent one cycle of PGT-A for gender selection due to X-linked disease. The results showed one normal female and one normal male embryo, while all remaining embryos were aneuploid. The couple decided to transfer together with the female embryo the male one, taking the risk that it had 50% chance of being affected. Prenatal diagnosis by chorionic villus sampling confirmed that the male embryo was unaffected and healthy twins (girl and boy) were born.

The contribution of PGT-M to prevent the transmission of severe and potentially fatal conditions and avoid miscarriages and implantation failure is positively acknowledged, leaving little moral controversy over the way conditions are classified as severe enough to be tested by PGT.In order for a genetic disorder to be classified as severe, simple identification of the mutation alone is inadequate. Other determining factors including impact on health, degree of penetrance (the potential for a mutated allele in the genotype to be expressed in the phenotype), therapy, hereditability, age of onset, and rate of progression should also be considered [11,26,27].

In addition, apart from the controversy over the determinants that classify a disorder as severe enough for an affected embryo to be deemed unfit for implantation, other ethical concerns arise. For instance, there is a possibility that individuals affected by a mutation accountable for a condition with incomplete penetrance eventually develop no symptoms. This possibility may lead to moral complications regarding the disposal of clinically affected embryos. Preimplantation genetic diagnosis can be applied to couples at high risk of developing multifactorial or late-onset diseases. In these cases, the method is close to the limits of eugenics as a genetic predisposition does not determine whether the disease manifests in the end. Concerns become apparent about the decision parents will make, as they find themselves in the dilemma of preventing the birth of such an embryo, which does not suffer and may never get sick, or eventually choosing to carry and give birth to an embryo with such predisposition and raise it accordingly, knowing this future danger.

According to the opinions contradicting PGT with HLA typing, the ethical complexity of the procedure regards the establishment of a “designer baby” era and the instrumentalization of the saviour child. Although the core purpose of PGT is to diminish the chance that a child affected by a severe genetic disease be born, in the case of HLA-matching, out of the total number of normal embryos created through IVF, one with the desired characteristic—HLA compatibility—is selected, in favour of every other embryo biopsied and disposed as healthy, but not HLA matched. In this particular aspect, the saviour child born has been exploited as a means, since its creation originally served a purpose. On the other hand, the nature of the purpose pursued is such that ultimately the management of the donor serves human values in the best way, since from the beginning of their life they will have been credited with the salvation of a fellow human being [17,18].

In general, PGT has been met with strict debate. More specifically, among the numerical chromosomal abnormalities diagnosed by PGT is trisomy 21 (Down syndrome), contributing to the ongoing discussion regarding discrimination against people born with the particular syndrome and the potential use of PGT as a eugenics tool, as well as presenting future parents with the dilemma to proceed with the implantation of an aneuploid embryo or discard it. Moreover, the accuracy of the biopsy itself to properly determine embryo ploidy has been challenged, since studies showed that mosaicism exists at all stages of preimplantation development, due to the lack of cell cycle check pointsleading to spindle and nuclear abnormalities [4,5,14,42,43]. It is important to note that low-medium mosaicism in the trophectoderm mostly arises after TE and ICM differentiation, and such embryos have equivalent developmental potential as fully euploid ones [44,45,46,47]. Mosaic embryos have the ability to implant, but it is uncertain whether they can go to full term. Mosaicism is a major factor affecting ongoing pregnancy rates but other factors including thrombophilia, infections that can lead to premature rapture of amniotic membranes, and immunological causes may also lead to miscarriages [4,8,20,22,42,47]. In cases of embryos analyzed only by PGT-M for mutations, the possibility of having aneuploidies too cannot be ruled out and this may also be a contributing factor affecting ongoing pregnancy rates. In the unfortunate event that a PGT-M pregnancy is complicated by additional aneuploidy, the pregnancy may end up in miscarriage or be terminated. Simultaneous analysis of both mutations and aneuploidies may be an attractive option but the increased cost in that case may be prohibitive. Currently in Greece, the cost for PGT-A is approximately 1900 euros for up to eight embryos and for PGT-M, 2000 euros including mutation analysis in patients’ blood and genetic counseling.

An important finding of the present study was the stress imposed on the couples, which overall, on a scale of 1 to 10, did not exceed 5; PGT was physically experienced notably more painful by the female partners, whereas the process had a higher psychological impact on the male ones. While there is rich literature regarding the emotional and physical stressors of IVF, such as anxiety, stress and depression, there is poor research specifically for PGT. The technical steps of the two processes are similar [28,29,30,31,32,33,34,35]. There is, however, a major difference regarding the added emotional burdens of couples undergoing PGT, related to family histories of genetic disorders [29]. One study regarding particularly PGT [28] interviewed 134 patients referred for PGT in the form of questionnaires; 55 deemed the experience extremely stressful; out of 20 patients who had undergone both prenatal diagnosis and PGT, 8 considered PGT less painful than prenatal diagnosis. The time during first consultation prior to a PGT cycle and the anticipation for a pregnancy result post embryo transfer were considered the most stressful stages of PGT treatment. Findings from other studies showed that oocyte retrieval can be a highly distressing period for women subjected to IVF in general [31,48]. A prospective study evaluating fluctuations in anxiety and distress levels in Australian women subjected to PGT [29] predicted that anxiety was dramatically increased post embryo transfer and following pregnancy test indication, with insignificant divergence in the distress experienced by women with a positive result from the ones with a negative one. Another aspect investigated by Lavery et al. [28] was the impact on the couples’ relationships; one third declared it affected the relationship with their partner negatively and one third indicated that it was a bonding experience for both of them. These findings suggest that PGT treatment can be experienced in different ways at different stages but can be deemed as an overall stressful process.

Patients with no normal embryos for transfer after PGT face the difficult situation of coming to terms with remaining childless or exploring other ways to parenthood including gamete donation or adoption. On top of that, the fate of the rejected embryos following PGT, whether to be simply discarded or donated for research raises further ethical dilemmas especially in cases of HLA matching in which genetically normal but not HLA-compatible embryos are not chosen for transfer as they cannot save the life of their sibling. However, those embryos can be a valuable source of embryonic stem cell research should the patients not consider them for clinical purposes or donate them to a childless couple. The bioethics and morality of human embryo research raises various concerns [49,50]. In our study, the majority of patients (90%) were keen to donate their chromosomally or genetically abnormal embryos for research instead of simply discarding them.

The sample of patients referred for PGT in Northern Greece in the present study is limited, both numerically and geographically, thus not allowing for a generalized conclusion. However, the diversity of the cases studied in such a small sample provides useful insight into the background of the couples choosing PGT.

## 5. Conclusions

PGT is an attractive alternative to prenatal diagnosis (PND) aiming to establishing a healthy pregnancy by identifying and avoiding the transfer of chromosomally or genetically abnormal embryos to the uterus. The fertilization, implantation, and ongoing pregnancy rates for the PGT-M and PGT-A cycles in the present study were 81.3%, 70.6%, and 52.9%, and 78.2%, 64.3%, and 57.1%, respectively. Although the benefits of PGT were well-received by all patients undergoing the procedure, psychological pain was evident and especially prominent in patients with a previous affected child or no normal embryos for transfer. Females experienced more intense physical pain than their male partners while psychological pain was encountered by both partners and occasionally in higher instances in males. No typical socioeconomic background of the patients referred for PGT in Northern Greece was noticed. Holistic counseling is of utmost importance in order to make patients’ experience during their journey to have a healthy baby less emotionally demanding and help them make the right choices for the future.

## Figures and Tables

**Table 1:** Clinical outcome of PGT-M cycles.

Preimplantation Genetic Testing Cycles (PGT-M) Day 3 Embryo Biopsy		19
Oocytes		252
MII Oocytes (mature)		214
2PN Zygotes		174 (81.3%)
Biopsied embryos (Day 3)		129
Normal		27
Carriers		41
Cycles WITHOUT embryos for transfer		2 (10.5%)
Cycles with embryos for transfer	Normal embryos for transfer	12/17
	Carrier embryos for transfer	5/17
+hCG/ET		12/17 (70.6%)
Ongoing pregnancy/ET		9/17 (52.9%)
Number of babies born: 10	Female	2/10
	Male	8/10

MII: Metaphase II Oocytes, 2PN: 2 pronuclei stage zygotes, ET: embryo transfer, hCG: human chorionic gonadotropin.

**Table 2:** Clinical outcome of PGT-Acycles.

Preimplantation Genetic Testing Cycles (PGT-A) Day 3 Embryo Biopsy/Array—CGHAnalysis		22
Oocytes		272
MII Oocytes (mature)		225
2PN Zygotes		176 (78.2%)
Biopsied embryos (Day 3)		126
Normal		25
Cycles WITHOUT embryos for transfer		8 (38.1%)
Cycles with normal embryos for transfer		14
+hCG/ET		9/14 (64.3%)
Ongoing pregnancy		8/14 (57.1%)
Number of babies born: 10	Female	4/10
	Male	6/10

MII: Metaphase II Oocytes, 2PN: 2 pronuclei stage zygotes, ET: embryo transfer, hCG: human chorionic gonadotropin, CGH: comparative genome hybridisation.

**Table 3:** Indications for PGT-M and PGT-A.

**Indications for PGT-M**	**Number of Cycles**
B-thalassemia	9
B-thalassemia and Micro-drepanocytosis	2
B-thalassemia and Sickle Cell Anemia	1
Cystic Fibrosis	1
X-Linked Chronic Granulomatous Disease (CYBB + HLA typing)	2
Duchenne Muscular Dystrophy	1
Metabolic disorder	1
Adenomatous polyposis coli (APC) gene	1
Breast Cancer (BRCA1)	1
Total	19
**Indications for PGT-A**	**Number of cycles**
AMA	3
RM	1
RIF	1
AMA and RIF	8
AMA and RM	6
X-Linked (gender selection)	3
Total	22

AMA: Advanced Maternal Age, RM: Repeated Miscarriages, RIF: Repeated Implantation Failure, CYBB: Cytochrome B-245 Beta Chain, HLA: Human Leucocyte Antigen.

**Table 4:** Binary logistic regression with respect to patient gender.

	B	S.E.	Wald	df	*p*_value_	Exp (B)	95% C.I. for EXP (B)	
							Lower	Upper
Psychological pain	0.309	0.161	3.662	1	0.056	1.362	0.993	1.868
Physical pain	−0.365	0.180	4.133	1	0.042	0.694	0.488	0.987
Constant	−1.324	0.983	1.816	1	0.178	0.266		

B: the values for the logistic regression equation for predicting the dependent variable from the independent variable.

**Table 5:** Sociodemographic characteristics of the patients undergoing PGT.

	Total	Men	Women
Age in years			
Mean	39.26	40.93	38
SD	4.62	4.23	4.6
Origin			
Greek	97.1%	100.0%	95.0%
Other	2.9%	0.0%	5.0%
Education			
Primaryorless	2.9%	6.7%	0.0%
Apprenticeship	0.0%	0.0%	0.0%
Secondary	34.3%	33.3%	35.0%
University	48.6%	46.7%	50.0%
MSc/PhD	14.3%	13.3%	15.0%
Employment			
Businessman/woman	5.7%	6.7%	5.0%
Private-sectoremployee	48.6%	53.3%	45.0%
Public-sectoremployee	25.7%	33.3%	20.0%
Freelancer	14.3%	6.7%	20.0%
Household	2.9%	0.0%	5.0%
Unemployment	2.9%	0.0%	5.0%
Religious affiliation			
Yes	94.0%	100.0%	90.0%
No	6.0%	0.0%	10.0%
Income			
−€10,000	11.4%	13.3%	10.0%
€10,000–€19,999	11.4%	13.3%	10.0%
€20,000–€29,999	34.3%	46.7%	25.0%
€30,000–€39,999	28.6%	20.0%	35.0%
€40,000–€50,000	8.6%	6.7%	10.0%
More than €50,000	0.0%	0.0%	0.0%

## Data Availability

The data presented in this study are available on request from the corresponding author.
